# Supplementary material for: A rapid workflow for neuron counting in combined light sheet microscopy and magnetic resonance histology
Source: Front Neurosci. 2023 Sep 27;17:1223226. doi: 10.3389/fnins.2023.1223226 (PMC10569694; doi:10.3389/fnins.2023.1223226)
Supplement: Supplementary file 1 [file Data_Sheet_1.docx]

**Supplemental**

**S1. Example of how to get training data for Random Forest**

**
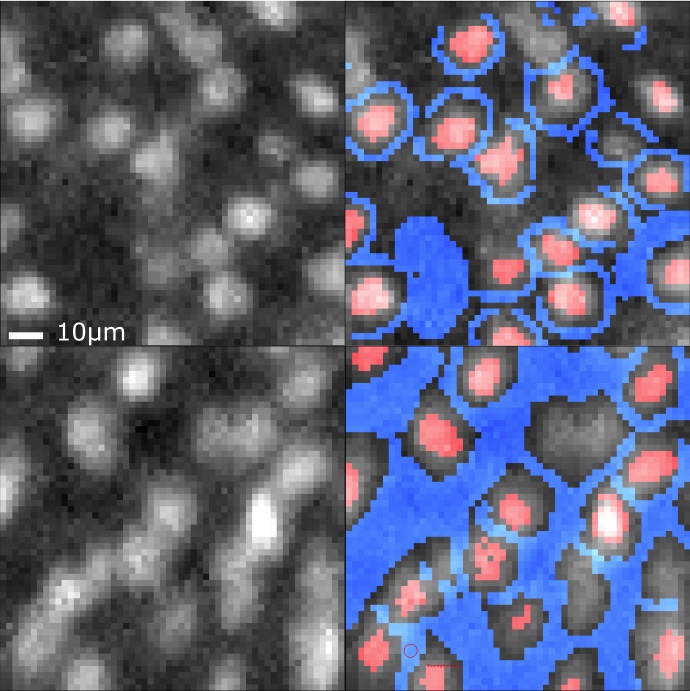
**

Figure S1 Left: Raw light sheet images from one of the subvolumes used in the counting algorithm. Right: Training data with blue sampling background neuropil and red labeled neuronal cell bodies stained with NeuN immunocyotchemistry. Typically, around 20 neurons need to be labeled with both the cell body and background for the algorithm to learn the accurate features of neurons (seen in the next section). The figure depicts two different scenarios. In the first row (auditory cortex), there are no overlapping neuronal cell bodies, and the background labels are applied between the neuronal cell bodies and the background neuropil. However, in the second row (field CA3), the image shows overlapping neuronal cell bodies. It's recommended to avoid adding a background label in the connected regions as it can lead to inaccurate classification. Instead, leave the connected neuron segmentation to watershed and volume filter.

**S2.F1 score, precision and recall change with the number of training nuclei**

To investigate the impact of the number of training examples, we utilized the pre-trained model with varied number of training nuclei, on multiple subvolumes within the selected region. This region was selected due to its dense neuronal population. Figure S2 provides a visualization of the F1, precision and recall scores as they change with the number of training examples.

F1 score, precision, and recall are commonly used metrics in classification tasks to evaluate the accuracy and performance of a model's predictions. Precision is the ratio of true positive predictions to the total predicted positive instances, measuring the accuracy of positive predictions. It is calculated as Precision = True Positives / (True Positives + False Positives).

Recall is the ratio of true positive predictions to the total actual positive instances, assessing the model's ability to identify positive instances. It is calculated as Recall = True Positives / (True Positives + False Negatives).

F1 Score is the harmonic mean of precision and recall, representing the balance between the two metrics and indicating overall model performance. It is calculated as F1 = 2 * (Precision * Recall) / (Precision + Recall).

In Figure S2, the training is on different number of nuclei examples and the validation subvolumes are distributed across the region as depicted in Figure S2 A. The masks generated by the machine segmentation are compared with the groundtruth manual labelling to obtain the scores. The results demonstrate that training on approximately 20 nuclei can yield an F score of around 0.9, while still maintaining flexibility and ease of use. These findings highlight the potential for achieving high accuracy in neuron counting with a relatively small number of training examples.
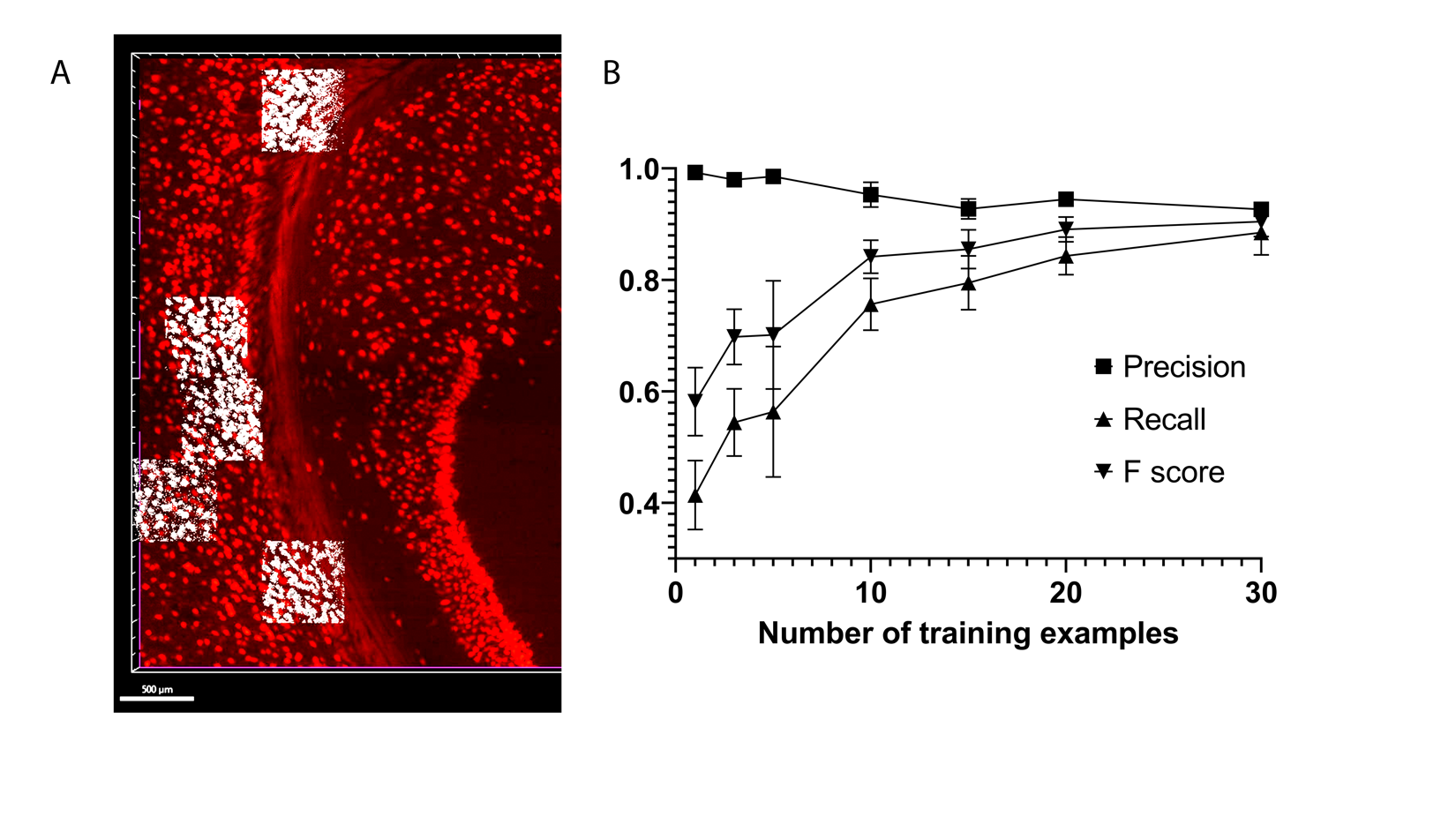


Figure S2 illustrates the location of the validation subvolumes and showcases the changes in scores corresponding to the number of training nuclei examples (each example is the individual nuclei and its surrounding background).

S3. **Simulation illustration of the watershed pipeline**

In order to gain a comprehensive understanding of the watershed function, we conducted a simulation utilizing spheres to effectively illustrate this component within the proposed workflow. The tif stack encompassing overlapping spheres was generated through Python's numpy library and subsequently underwent processing involving the watershed portion of the workflow within FIJI.


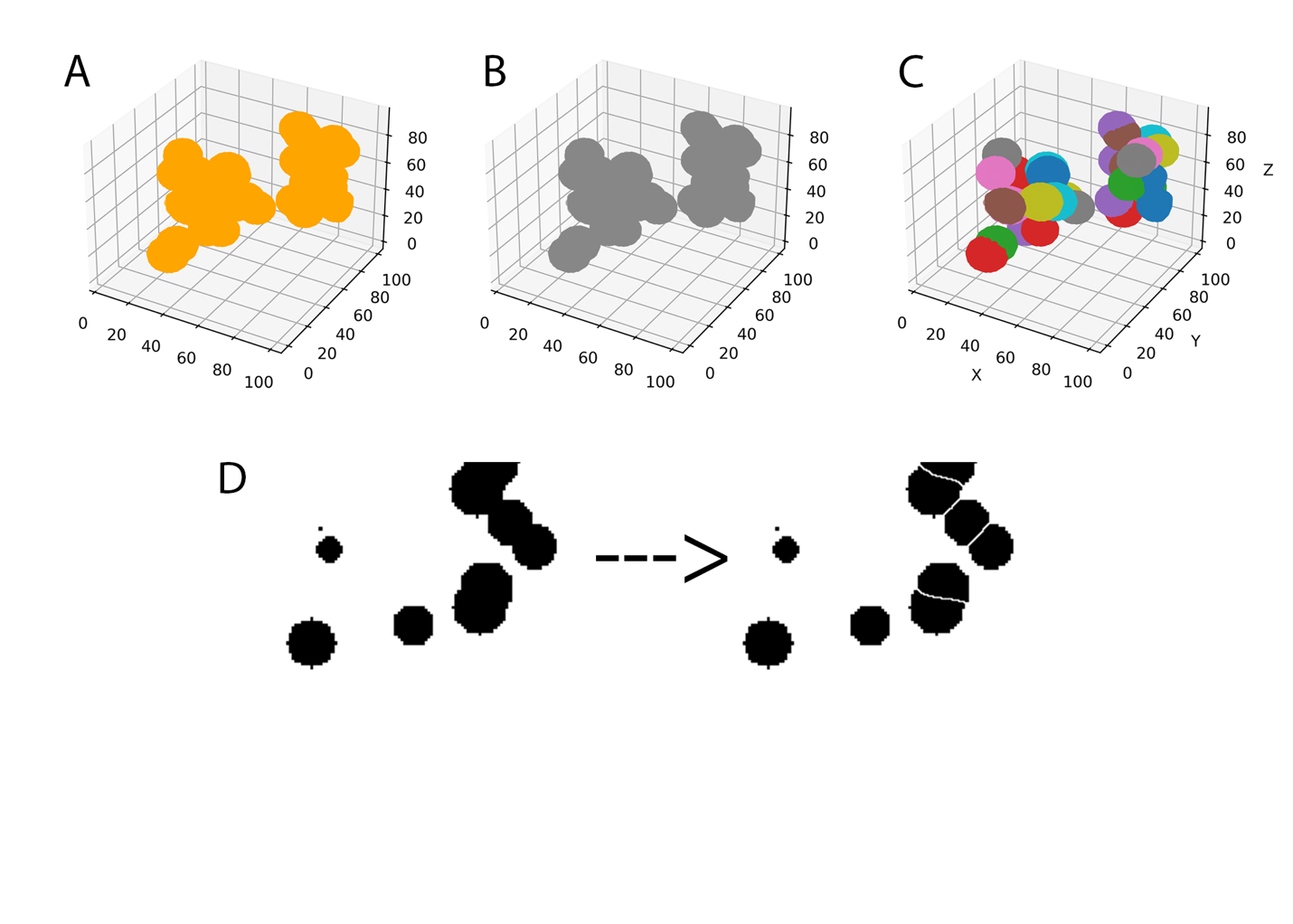


Figure S3 The simulation for watershed. Panel A, a simulation showcases 30 heavily overlapping spheres with sizes ranging from 8 to 10 um, confined within a 100um cube. Within our practical workflow, neuronal segmentation is executed to isolate neurons from the background, resulting in a distinct binary map, as simulated in Panel B. Furthermore, Panel C portrays the processed image post binarization, 3D watershed transformation, and 'Connected Components Labeling' operations carried out using the macro, which segregates the spheres and assigns a unique value to each. Random color assignment was undertaken in Python. The impact of the watershed operation is elucidated through a cross-sectional view of the simulated cube in Panel D.

**S4.Neuron density acquired from raw and corrected LSM**


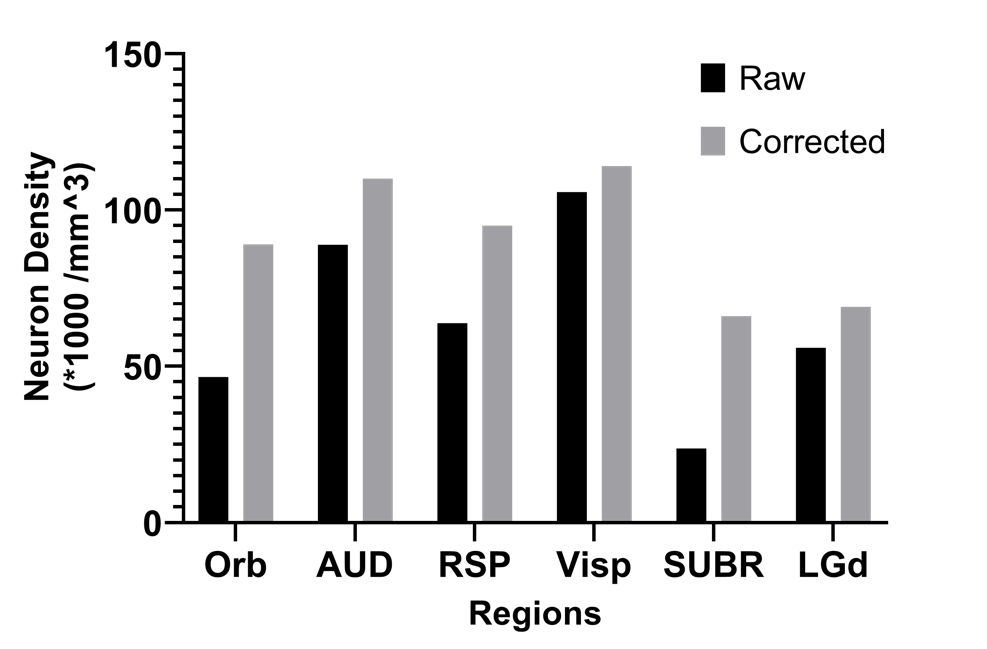


Figure S4 shows the comparison of neuron density between raw LSM data and deformation-corrected LSM data for six brain regions. Specimen: 200316.

**S5.Table S1 Comparison with other works**

Figure S4 and Table S1 present a comparison between the neuron density measurements obtained from our workflow and those reported in other studies of mouse brain. As our workflow reports neuron density, we converted other studies' reported neuron numbers (if any) to neuron density by dividing them with the corresponding CCFv3 volume. Our results are generally consistent with those of other studies, but in some regions, such as LGd, TH, BLA, we observe higher neuron density. This difference may be attributed to the fact that we removed tissue swelling during LSM data pre-processing. In regions with high heterogeneity in neuron distribution, such as subiculum and ENT, different studies report varying neuron density values, and our results fall within that range. This suggests that our results cover subregions with both sparse and dense neuron distribution in these regions. The findings are illustrated in Figure S1.

| Region | Reference | (Kuronen, Lehesjoki et al. 2012) | (Dursun, Jakubowska-Dogru et al. 2011) | (Scott, Jeffrey et al. 1994) | (Seecharan, Kulkarni et al. 2003) | (Ero, Gewaltig et al. 2018) | This work |
| --- | --- | --- | --- | --- | --- | --- | --- |
| Dorsal lateral geniculate nucleus | **Neuron density(no/**$\boldsymbol{mm}^{\boldsymbol{3}}$**)** | 55965 | 44517 | 38158 | 66333 | 62465 | 73200 |
|  | **Reference** | (Trujillo-Estrada, Davila et al. 2014) | (Fabricius, Wortwein et al. 2008) | (Ero, Gewaltig et al. 2018) |  |  |  |
| Subiculum | **Neuron density(no/**$\boldsymbol{mm}^{\boldsymbol{3}}$**)** | 155500 | 46965 | 83717 |  |  | 71860 |
|  | **Reference** | (Herculano-Houzel, Watson et al. 2013) | (Ero, Gewaltig et al. 2018) |  |  |  |  |
| Entorhinal areas | **Reference** | 66988 | 91110 |  |  |  | 80640 |
|  | **Neuron density(no/**$\boldsymbol{mm}^{\boldsymbol{3}}$**)** | (Herculano-Houzel, Watson et al. 2013) | (Ero, Gewaltig et al. 2018) |  |  |  |  |
| Auditory | **Neuron density(no/**$\boldsymbol{mm}^{\boldsymbol{3}}$**)** | 109,730 | 108440 |  |  |  | 105805 |
|  | **Reference** | (Herculano-Houzel, Watson et al. 2013) | (Ero, Gewaltig et al. 2018) |  |  |  |  |
| Retroplenial | **Neuron density(no/**$\boldsymbol{mm}^{\boldsymbol{3}}$**)** | 98148 | 101650 |  |  |  | 84479 |
|  | **Reference** | (Herculano-Houzel, Watson et al. 2013) | (Ero, Gewaltig et al. 2018) |  |  |  |  |
| Orbital | **Neuron density(no/**$\boldsymbol{mm}^{\boldsymbol{3}}$**)** | 48109 | 77961 |  |  |  | 78333 |
|  | **Reference** |  | (Ero, Gewaltig et al. 2018) |  |  |  |  |
| Facial motor nucleus | **Neuron density(no/**$\boldsymbol{mm}^{\boldsymbol{3}}$**)** |  | 36427 |  |  |  | 9700 |
|  | **Reference** |  | (Ero, Gewaltig et al. 2018) |  |  |  |  |
| trigeminal | **Neuron density(no/**$\boldsymbol{mm}^{\boldsymbol{3}}$**)** |  | 45986 |  |  |  | 58607 |
|  | **Reference** | (Fabricius, Wortwein et al. 2008) | (Ero, Gewaltig et al. 2018) | (Hlatky, Lui et al. 2003) | (Liu, Yu et al. 2006) |  |  |
| CA1 | **Neuron density(no/**$\boldsymbol{mm}^{\boldsymbol{3}}$**)** | 44500 | 96289 | 52000 | 38290 |  | 80293 |
|  | **Reference** | (Fabricius, Wortwein et al. 2008) | (Ero, Gewaltig et al. 2018) | (Hlatky, Lui et al. 2003) |  |  |  |
| CA3 | **Neuron density(no/**$\boldsymbol{mm}^{\boldsymbol{3}}$**)** | 53900 | 135494 | 88000 |  |  | 112233 |
|  | **Reference** |  | (Ero, Gewaltig et al. 2018) |  |  |  |  |
| Primary visual | **Neuron density(no/**$\boldsymbol{mm}^{\boldsymbol{3}}$**)** |  | 100245 |  |  |  | 115343 |
|  | **Reference** |  | (Ero, Gewaltig et al. 2018) |  |  |  |  |
| Thalamus | **Neuron density(no/**$\boldsymbol{mm}^{\boldsymbol{3}}$**)** |  | 78611 |  |  |  | 108200 |
|  | **Reference** |  | (Ero, Gewaltig et al. 2018) | (Mozhui, Hamre et al. 2007) |  |  |  |
| BLA | **Neuron density(no/**$\boldsymbol{mm}^{\boldsymbol{3}}$**)** |  | 77920 | 74375 |  |  | 87386 |

Table S1. The comparison of neuron density across literature and from our reported workflow.


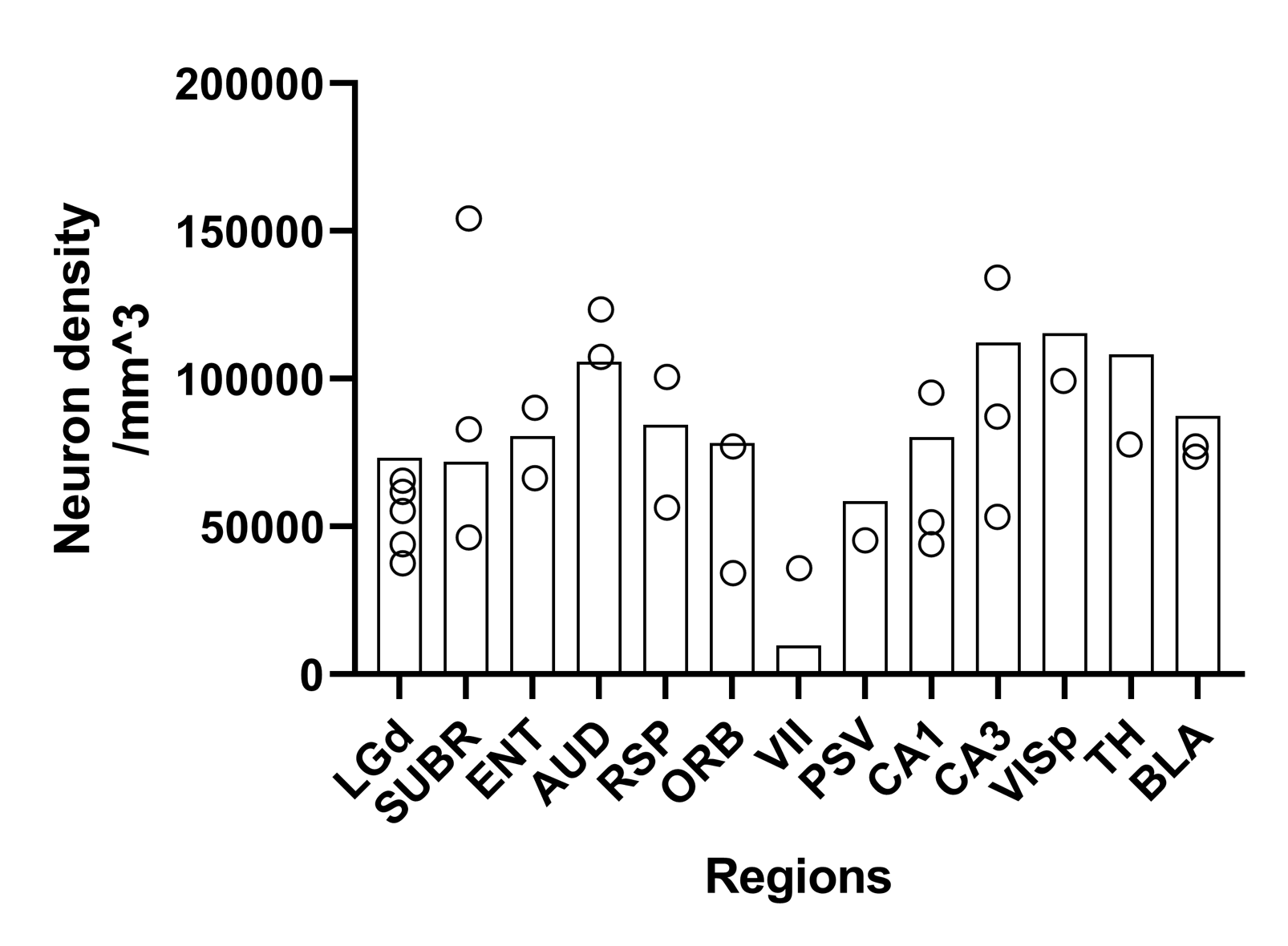


Figure S5 presents a comparison of neuron density values obtained from our workflow with those reported in the literature. The figure highlights any differences between our findings and those of other studies in different brain regions. The bar represents the values obtained from our workflow, while the circles indicate the corresponding values reported in peer studies.

**S5.1 Calculation of neural density across literature**

The literature search was conducted using keywords related to brain regions and their broader categories. The databases used were Google Scholar and PubMed. For example, in Entorhinal cortex, the keyword is below:

neuron* AND (densit* OR population* OR number* OR cell atlas) AND ("entorhinal cortex" OR EC OR allocortex) AND ("mice" OR "mouse")

During the literature search, if a study uses methods such as isotropic fractionator (IF) or stereology, and reports both the neuron countand the neuron density, we will divide the reported neuron count by the corresponding CCFv3 volume.

Regarding subject strains, age, and sex, these factors can have an impact on neuron density. If a study presents the neuron density or number indirectly, we will provide further explanation. However, if a study does not report age or sex, no mention will be made in the explanation.

**LGd:** In the first three references, the neuron numbers are approximated from the figures they provided for the wild type (WT, C57BL/6J).

In (Kuronen, Lehesjoki et al. 2012), the number is the average of WT 1 month and 5 month.

In (Dursun, Jakubowska-Dogru et al. 2011), the number is from newborn control group.

In (Scott, Jeffrey et al. 1994), the number is from 100 days control group.

In (Seecharan, Kulkarni et al. 2003), age is not specified for 8 C57BL/6J mice, but for the large herd of mice, the average age is 100d and sex ratio is 1:1. The neuron number is obtained from Table1.

As explained in main text, (Ero, Gewaltig et al. 2018) does not include the measurement but instead employs the whole brain cell counts and monte carlo method to approximate the distribution. The blue brain map from (Ero, Gewaltig et al. 2018) provides the density.

**Subiculum:** In (Trujillo-Estrada, Davila et al. 2014):

The neuron density is obtained from the equation: Neuron density ~= density of SoM-interneurons + PV-interneurons (inhibitory neurons) + Principal neurons (excitatory neurons). Therefore, the neuron density is approximated from the figures:

2 month WT (C57BL/6J) ~= 17000 + 10500 + 128000 ~ 155500 neurons/mm^3

6 month WT (C57BL/6J) ~= 12000 + 10500 + 120000 ~ 142500 neurons/mm^3

In (Fabricius, Wortwein et al. 2008): The neuron number is obtained from optical fractioner, and therefore we have to use the subvolume they provide to get the density. Control group (C57BL/6J 2month 4 male and 8 female) mean neuron number is 181. Volume is obtained from Cavelieri principles. Volume = 2.51mm^2 x section thickness = 2.51mm^2 x 39um. The density is 46965/mm^3.

In (Ero, Gewaltig et al. 2018): They approximated the neural density from the observed density by a transfer function. The neuron density of subiculum provided in supplemental is 83717/mm^3.

**ENT, AUD, RSP and ORB**: in these cortical regions, (Herculano-Houzel, Watson et al. 2013) provides the bias-free neuron numbers generated from IF. The neuron density is obtained from dividing the neuron number by the CCFv3 regional volume. The subjects are four male C57BL/6J mice aged 6 weeks.

RSP: 314,761/5.5147 ~ 57076 /mm^3

ENT: 400019/5.9712 ~ 67000/mm^3

AUD: 377362/3.03 ~ 124542/mm^3

ORB: 107179/3.09 ~ 34685 /mm^3

**CA1, CA3:** In (Fabricius, Wortwein et al. 2008), subjects are C57BL/6J 2month 4 male and 8 female. Figure 8 provides the neuron number. We divide the number with CCFv3 volume.

In (Hlatky, Lui et al. 2003), the wild type C57BL/6J subjects (male, age unspecified) provide the neuron density.

**CA1:** (Liu, Yu et al. 2006) SAMR1 (male, control, resistant to early senescence of age-related disease) mice in this study yield CA1 neuron number 206,000 ± 11,000 neurons in 4-month-old and 190,000 ± 10,000 in 8-month-old.

Density = 206000/CCF volume 5.38 = 38290 /mm^3 (4-month old)

BLA: (Mozhui, Hamre et al. 2007) we take the neuron number from C57BL/6J subjects. The average age is 96 days and the range is from 30 to 500 days. The sex ration is 96 (F) : 103 (M).

**S6.Comparison with other software**

We selected Random Forest as our method due to its low requirement for training data and computational efficiency. During our evaluation, we considered alternative approaches such as 3D segmentation using the deep learning-based CellPose Model (Stringer, Wang et al. 2021), which offers the convenience of pretrained neuron network models. The CellPose Model took an average of around 1500 seconds to run the network and generate masks for a dataset of size 250 x 250 x 10 um (500 x 500 x 20 voxels) under cyto mode. The resulting masks exhibited relatively low F1 scores, likely due to the model being pretrained on a dataset that differed significantly from our test dataset.

For StarDist, we utilized the StarDist2D method (Schmidt, Weigert et al. 2018) and applied it slice by slice. Consequently, the scores and computational time may vary from those of 3D segmentation. Compared to these two methods, which require extensive pretraining and assume that neurons share similar characteristics with the training data, our proposed method demonstrates greater flexibility and computational efficiency.

In this comparison, we opted not to employ GPU due to the relatively small size of the subvolumes in practice.

|  | CellPose | StarDist2D | Proposed method |
| --- | --- | --- | --- |
| F1 score | 0.69 | 0.85 | 0.89 |
| Computing time | ~1500 s | ~15s | <5s |

Table S2 The F scores and computing times for different methods. The timing recorded for each method corresponds to the time taken for running the models and iterating through the slices in the case of the 2D method.


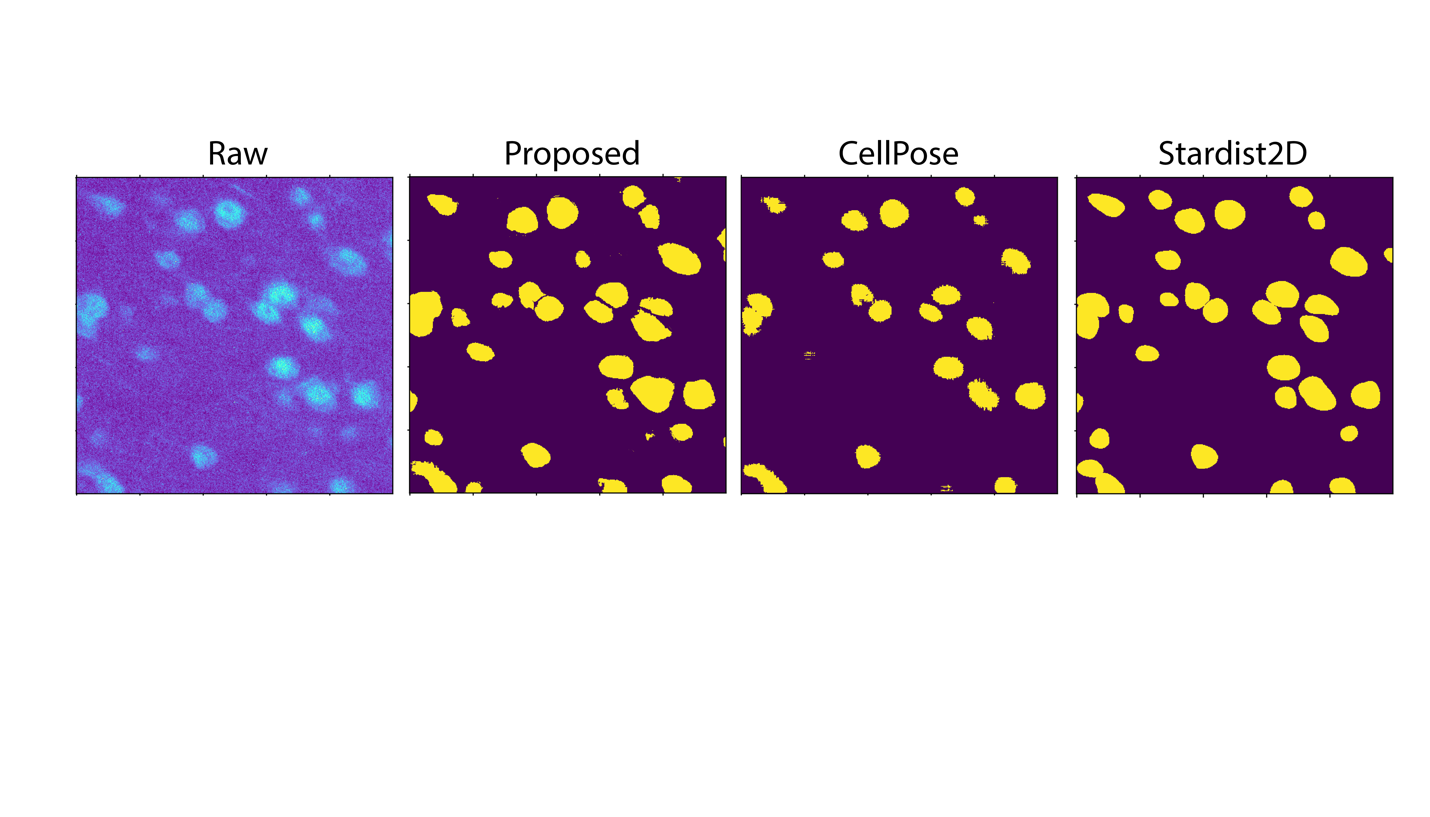


Figure S6 showcases the masks generated using different methods, all of which utilized pre-trained models.

**S7.Impact of Image quality on segmentation**

The performance of our method is highly dependent on image quality. In cases where neurons are densely compacted or the background signal is high or the specific staining signal is low, researchers may struggle to segment the individual neuronal cell bodies. Unfortunately, some of the data used in our study suffers from these issues. To address this problem, our workflow includes a compromised approximation that estimates the blob's volume over the neuron volume when segmentation is challenging. This approximation is based on the median neuron volume from the training data. Figure S2 presents examples of cases where our workflow was unable to accurately segment neurons due to poor image quality and densely compacted neurons.

To avoid this issue, researchers can use higher quality data, such as higher resolution data from expansion microscopy or LSM acquired with a higher power objective. However, this solution may not fully address the problem, particularly in regions where neurons are densely packed.


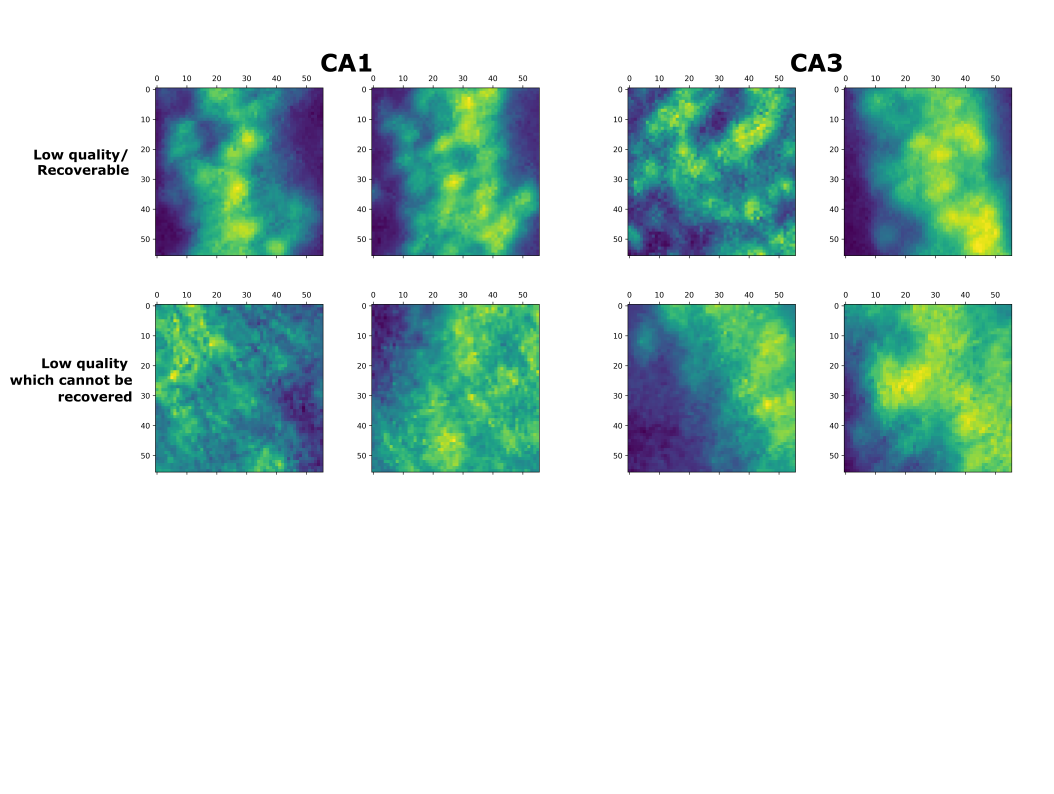


Figure S7 provides examples of low-quality images due to densely stacked neurons or poor imaging conditions, taken from CA1 and CA3. The first row shows an image that can be counted using the approximation, while the second row shows an image with such poor quality that it cannot be recovered. The side scale shows the number of pixels, and each image measures 100 μm x 100 μm

S8.Coefficient of variation across animals

| Regions | Neuron density  (mean CV +/- SD) | Neuron number  (mean CV +/- SD) |
| --- | --- | --- |
| LGd | 0.11 | 0.13 |
| AUD | 0.08 | 0.08 |
| RSP | 0.07 | 0.06 |
| Orb | 0.09 | 0.07 |
| SUBR | 0.10 | 0.09 |
| VII | 0.33 | 0.35 |
| PSV | 0.24 | 0.44 |
| ENT | 0.15 | 0.13 |
| CA1 | 0.06 | 0.08 |
| CA3 | 0.09 | 0.07 |
| VISp | 0.15 | 0.13 |
| TH | 0.17 | 0.16 |
| BLA | 0.30 | 0.27 |

Table S3 shows the coefficient of variation across animals.

S9.Sampling ratio impact on the output

According to the pioneering gold standard stereology paper (Page 26, between equation 17 and 18 in (Gundersen and Jensen 1987) ), stereologists typically use a sampling rate of the order of 1/1000. In our demonstration, we employed 15 subvolumes of size 100 um x 100 um x 100 um to sample the entire region, resulting in a sampling ratio ranging from 0.25% to 4%, which is well above the 1/1000 threshold.

While increasing the sampling ratio in stereology can be time-consuming due to the need to manually slice and count, our method allows for easy adjustment of the sampling ratio by either increasing the number of subvolumes or their size. Both adjustments can be done effortlessly by altering the parameters. To further illustrate the influence of the sampling ratio on the final density counts, we have conducted a simple experiment done in Auditory region of one specimen, the results of which are presented in this section.

| Sampling ratio | 0.25% | 0.5% | 0.75% | 1% | 5% |
| --- | --- | --- | --- | --- | --- |
| Mean (*10^3 /mm^3) | 103.267 | 99.967 | 100.04 | 99.0167 | 101.47 |
| STD (*10^3 /mm^3) | 14.74 | 18.77 | 18.049 | 14.51 | 18.1 |

Table S4 presents the density output obtained from measurements at various sampling ratios.

This experiment demonstrates that increasing the sampling ratio does not significantly impact the mean density. The variation in standard deviation observed can be attributed to the inherent heterogeneity introduced by random sampling. Overall, the density output remains stable whether we sample 15, 60, or 300 subvolumes.

**Reference:**

Dursun, I., E. Jakubowska-Dogru, D. van der List, L. C. Liets, J. L. Coombs and R. F. Berman (2011). "Effects of early postnatal exposure to ethanol on retinal ganglion cell morphology and numbers of neurons in the dorsolateral geniculate in mice." Alcohol Clin Exp Res **35**(11): 2063-2074.

Ero, C., M. O. Gewaltig, D. Keller and H. Markram (2018). "A Cell Atlas for the Mouse Brain." Front Neuroinform **12**: 84.

Fabricius, K., G. Wortwein and B. Pakkenberg (2008). "The impact of maternal separation on adult mouse behaviour and on the total neuron number in the mouse hippocampus." Brain Struct Funct **212**(5): 403-416.

Gundersen, H. J. and E. B. Jensen (1987). "The efficiency of systematic sampling in stereology and its prediction." J Microsc **147**(Pt 3): 229-263.

Herculano-Houzel, S., C. Watson and G. Paxinos (2013). "Distribution of neurons in functional areas of the mouse cerebral cortex reveals quantitatively different cortical zones." Front Neuroanat **7**: 35.

Hlatky, R., H. Lui, L. Cherian, J. C. Goodman, W. E. O'Brien, C. F. Contant and C. S. Robertson (2003). "The role of endothelial nitric oxide synthase in the cerebral hemodynamics after controlled cortical impact injury in mice." J Neurotrauma **20**(10): 995-1006.

Kuronen, M., A. E. Lehesjoki, A. Jalanko, J. D. Cooper and O. Kopra (2012). "Selective spatiotemporal patterns of glial activation and neuron loss in the sensory thalamocortical pathways of neuronal ceroid lipofuscinosis 8 mice." Neurobiol Dis **47**(3): 444-457.

Liu, C. Z., J. C. Yu, H. Y. Cheng, Z. G. Jiang, T. Li, X. Z. Zhang, L. L. Zhang and J. X. Han (2006). "Spatial memory performance and hippocampal neuron number in osteoporotic SAMP6 mice." Exp Neurol **201**(2): 452-460.

Mozhui, K., K. M. Hamre, A. Holmes, L. Lu and R. W. Williams (2007). "Genetic and structural analysis of the basolateral amygdala complex in BXD recombinant inbred mice." Behav Genet **37**(1): 223-243.

Schmidt, U., M. Weigert, C. Broaddus and G. Myers (2018). "Cell Detection with Star-Convex Polygons." Medical Image Computing and Computer Assisted Intervention - Miccai 2018, Pt Ii **11071**: 265-273.

Scott, J. R., M. Jeffrey and W. G. Halliday (1994). "Unsuspected early neuronal loss in scrapie-infected mice revealed by morphometric analysis." Ann N Y Acad Sci **724**: 338-343.

Seecharan, D. J., A. L. Kulkarni, L. Lu, G. D. Rosen and R. W. Williams (2003). "Genetic control of interconnected neuronal populations in the mouse primary visual system." J Neurosci **23**(35): 11178-11188.

Stringer, C., T. Wang, M. Michaelos and M. Pachitariu (2021). "Cellpose: a generalist algorithm for cellular segmentation." Nat Methods **18**(1): 100-106.

Trujillo-Estrada, L., J. C. Davila, E. Sanchez-Mejias, R. Sanchez-Varo, A. Gomez-Arboledas, M. Vizuete, J. Vitorica and A. Gutierrez (2014). "Early neuronal loss and axonal/presynaptic damage is associated with accelerated amyloid-beta accumulation in AbetaPP/PS1 Alzheimer's disease mice subiculum." J Alzheimers Dis **42**(2): 521-541.
